# Analysis of Unweighted Amino Acids Network

**DOI:** 10.1155/2014/350276

**Published:** 2014-12-16

**Authors:** Adil Akhtar, Tazid Ali

**Affiliations:** Department of Mathematics, Dibrugarh University, Dibrugarh, Assam 786004, India

## Abstract

The analysis of amino acids network is very important to studying the various physicochemical properties of amino acids. In this paper we consider the amino acid network based on mutation of the codons. To analyze the relative importance of the amino acids we have discussed different measures of centrality. The measure of centrality is a powerful tool of graph theory for ranking the vertices and analysis of biological network. We have also investigated the correlation coefficients between various measures of centrality. Also we have discussed clustering coefficient as well as average clustering coefficient of the network. Finally we have discussed the degree of distribution as well as skewness.

## 1. Introduction

Amino acids are the building blocks of proteins. Each protein is formed by a linear chain of amino acids. There are 20 different amino acids being found till now that occur in proteins. Each amino acid is a triplet code of four possible bases. A sequence of three bases forms a unit called codon. A codon specifies one amino acid. The genetic code is a series of codons that specify which amino acids are required to make up specific protein. As there are four bases, (Adenine (A), Cytosine (C), Guanine (G), or Thymine (T/U)) this gives us 64 codons. Out of these 64, the three triplets UAA, UAG, and UGA are known as stop codons or nonsense codons and their role is to stop the biosynthesis. The codon AUG codes for the initiation of the translation process and is therefore also known as start codon. Also a codon can be changed in several ways; such change is known as mutation. There are various types of mutation like substitution, insertion, deletion, frameshift, and so forth. In this paper we have considered one-point mutation of all possible bases. To discuss relative importance or significance of amino acids we have investigated four centrality measures in the amino acid network. The compatibility relation of the graph is defined based on the mutation of the codon. For example the amino acid M (Methionine) is connected with K (Lysine), T (Threonine), R (Argnine), I (Isoluecine), V (Valine), L (Leucine), because all possible mutations of the base of the codon AUG (M) represent amino acids K, T, R, I, V and L. Different researchers have made many contributions in this field. Kundu [1] discussed that hydrophobic and hydrophilic network satisfy “small-world property” within protein. Also he has discussed that hydrophobic network has large average degrees of nodes than the hydrophilic network. In 2007 Aftabuddin and Kundu [2] discussed three types of networks within protein and give some idea about all three types of networks. Jiao et al. [3] discussed the weighted amino acid network based on the contact energy. They have shown that weighted amino acid network satiety is “small-world” property. Fell and Wagner [4] examined whether metabolites with highest degree may belong to the oldest part of the metabolism. Wuchty and Stadler [5] discussed various centrality measures in biological network. They concluded that the degree of vertex centrality alone is not sufficient to distinguish lethal protein from viable ones. Newman [6] discussed correlation of degree of centrality and betweenness centrality. Also Schreiber and Koschutzki [7] compared centralities for biological networks, namely, PPI network and transcriptional network. As a result of their study, it was observed that in the analysis of biological networks various centrality measures should be considered.

This paper is organized as follows. In Section 2 we define some preliminary concepts of the graph on which we operate and briefly review the various centrality measures. In Section 3 we define graph in amino acids based on mutation and discuss various centrality measures. Also we discuss the bivariate correlation between different centrality measures. In Section 4 we discuss some network parameters. In Section 5 we give the conclusion of this paper.

## 2. Preliminary Concepts of Graph

An undirected graph *G* = (*V*, *E*) consists of a finite set *V* of vertices and a finite set *E*⊆*V* × *V* of edges. If an edge *e* = (*u*, *v*) connects two vertices *u* and *v* then vertices *u* and *v* are said to be incident with the edge *e* and adjacent to each other. The set of all vertices which are adjacent to *u* is called the neighborhood *N*(*u*) of *u*. The complete graph is a graph in which each of the vertices connects to one another. A directed graph or digraph *G* consists of a set *V* of vertices and a set *E* of edges such that *e* ∈ *E*, if each edge of the graph *G* has a direction. A graph is called loop-free if no edge connects a vertex to itself. An adjacency matrix *A* of a graph *G* = (*V*, *E*) is a (*n* × *n*) matrix, where *a*_*ij*_ = 1 if and only if (*i*, *j*) ∈ *E* and *a*_*ij*_ = 0 otherwise. The adjacency matrix of any undirected graph is symmetric. The degree of a vertex *v* is defined to be the number of edges having *v* as an end point. A walk is defined as a finite alternating sequence of vertices and edges, beginning and ending with vertices, such that each edge is incident with the vertices preceding and following it. No edges appear more than once in a walk. A vertex, however, may appear more than once. In a walk beginning and ending vertices are initial and terminal vertices. A walk is closed if beginning and end vertices are the same. Also if beginning and end vertex are not the same then that walk is called open walk. A trail is a walk without repeated edges and path is a walk without repeated vertices. A shortest or geodesic path between two vertices *u*, *v* is a path with minimal length. A graph is connected if there exists a walk between every pair of its vertices.

### 2.1. Centrality in Graph

In graph theory, centrality measure of a vertex represents its relative importance within the graph. A centrality is a real-valued function on the nodes of a graph. More formally a centrality is a function *f* which assigns every vertex *v* ∈ *V* of a given graph *G* a value *f*(*v*) ∈ *R*. In the following we have discussed four most commonly used centrality measures.

#### 2.1.1. Degree of Centrality

The most simple centrality measure is degree of centrality, *c*_*d*_(*u*). It is defined as the number of nodes to which the node *u* is directly connected. The nodes directly connected to a given node *u* are also called first neighbors of the given node. Degree centrality shows that an important node is involved in a large number of interactions. This interaction gives the immediate importance or risk of the node in the corresponding network. Mathematically it is defined as

(1) $$\begin{array}{c} c_{d}\left(u\right)=\deg\left(u\right). \end{array}$$

However in real world problem the degree of centrality is not an actual measurement for finding importance or risk of a node. In real situation an important node may be connected indirectly with other nodes.

#### 2.1.2. Eigenvector Centrality

Another important measure of centrality is eigenvector centrality [8]. An eigenvalue of a square matrix *A* is a value *λ* for which det⁡(*A* − *λI*) = 0, where *I* is the identity matrix of the same order as *A*. Eigenvector centrality is defined as the principal eigenvector of the adjacency matrix of corresponding graph.

In matrix-vector notation we can write

(2) $$\begin{array}{c} \lambda X=AX, \end{array}$$

where *A* is the adjacency matrix of the graph, *λ* is a constant (the eigenvalue), and *X* is the eigenvector. In general, there will be different eigenvalues *λ* for which an eigenvector solution exists. However eigenvector of the greatest eigenvalue is the eigenvector centrality [8]. Eigenvector centrality gives the direct as well as indirect importance of a node in a network.

#### 2.1.3. Closeness Centrality

The closeness centrality is the idea how a vertex is close to all other vertices not only to the first neighbor but also in global scale. Generally a vertex is central; then it is close to all other vertices. If a vertex is close to other vertices, then it can quickly interact with all other vertices. In general closeness centrality is defined as the inverse of the sum of the shortest path distances between each node and every other node in the network [9]. The closeness centrality of a node depicts an important node that can easily reach or communicate with other nodes of the network. Mathematically it is defined as

(3) $$\begin{array}{c} C_{\mathrm{cl}}\left(u\right)=\frac{\left(n-1\right)}{\sum_{v\in V}^{}d\left(u,v\right)}, \end{array}$$

where *n* is the number of vertices of the network and *d*(*u*, *v*) is the shortest path distance between the pair of vertices *u* and *v*. From the above definition it is clear that if a node has minimum cumulative shortest path distance, then that node has maximum closeness centrality. And maximum closeness centrality node is very well connected to all other nodes.

#### 2.1.4. Betweenness Centrality

Another well-known centrality measure is the betweenness centrality [9]. Betweenness centrality interactions between two nonadjacent nodes depend on the other node, generally on those on the paths between the two. The betweenness centrality of a node *u* is the number of shortest paths going through *u*. Mathematically it is defined as

(4) $$\begin{array}{c} C_{\text{btw}}\left(u\right)=\underset{s\neq u\in V}{\sum }\;\underset{t\neq u\in V}{\sum }\frac{\sigma_{\text{st}}\left(u\right)}{\sigma_{\text{st}}}, \end{array}$$

where *σ*_st_ is the number of shortest paths from vertex *s* to *t* and *σ*_st_(*u*) is the number of shortest paths from *s* to *t* that pass through *u*. Betweenness centrality depicts identifying nodes that make most information flow of the network. An important node will lie on a large number of paths between other nodes in the network. From this node we can control the information of the network. Without these nodes, there would be no way for two neighbors to communicate with each other. In general the high degree node has high betweenness centrality because many of the shortest paths may pass through that node. However a high betweenness centrality node need not always be high degree node.

## 3. Graph of Amino Acids

**Figure 1:**
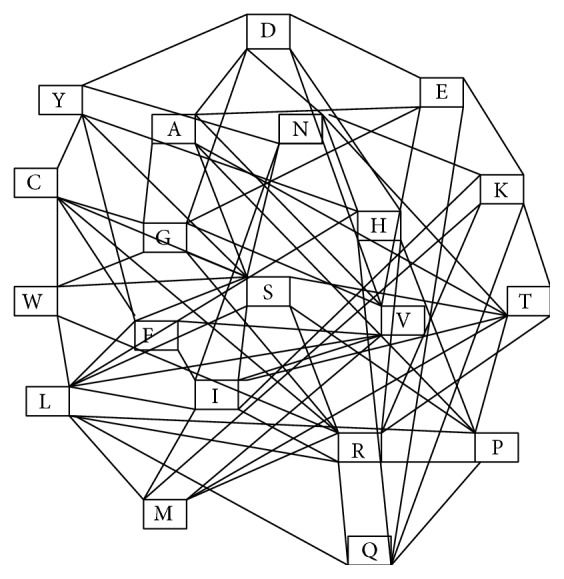


Every codon codes unique amino acids. A one-point mutation of a codon may or may not change the corresponding coded amino acid. All one-point mutations of a codon give nine more codons. Some of these nine codons will code for the same amino acid(s) other than the original one. In some sense the nine mutants can be termed near or close to the original one. In the language of topology these codons can be termed vicinity of the original codon. In other words they are related to the original one. Since any mutation has its reverse mutation, this relation is bidirectional. This nearness relation or affinity is naturally carried over to the amino acids. Thus in the amino acids we have a binary relation which generated an undirected graph. Thus in our amino acid graph the vertex set is the set of amino acids and two amino acids *α* and *β* are linked/connected by an edge if one-point mutation of a codon coding *α* codes for *β*. Thus two amino acids connected by an edge can be interpreted as having affinity towards each other in the sense that one may evolve from the other. Thus the amino acid graph gives a picture of the evolution of the amino acids. We will call it the evolutionary graph of amino acids. The corresponding graph is depicted in Figure 1.

From Figure 1, we observe that the graph is connected. Corresponding adjacency matrix of the graph is given as follows:

(5) M=0110100000101100101010100000011110001000110101110000100010000010011000010000001110000100001001000010001110110110000101100011010100110010001100100110101001000000000000010010011011000100010000110001011011001111110101100010010100100110001000111110000000000001100110011001101000000010000000101011000011010000010001001000011111100000100010100100000001001100001110101001111001110001010100010010000110110010.

### 3.1. Centralities in Amino Acids Graph

To analyse the amino acid graph (Figure 1) we have calculated different measures of centrality. In Table 1, we represent the different centrality values of the vertices.

From Figure 1 we observed that the graph is not complete. That means some of the amino acids are not linked with some other amino acids. The amino acids R (Arginine) and S (Serine) form a complete graph with respect to first base or third base mutation of codon. Again the amino acids R and S are well connected to all other amino acids through first base and/or second base mutations so its cumulative shortest path distance is minimum. Hence the amino acids R and S have high closeness centrality. Therefore the first base and/or second base mutation has relative importance in terms of closeness centrality.

Further we observed that any amino acid which has no direct link with other amino acids, has indirect link through one of R or S. For example, the amino acid G has no direct link with the amino acids, namely, M, L, I, F, Y, P, T, N, Q, K, and H. But through the amino acids R and S with the help of first base and/or second base mutation they are linked indirectly. Again when we observe betweenness centrality it is clear that the amino acids R and S have high betweenness value. Because the degree of these amino acids is high, many shortest paths pass through them. As R and S are linked with other amino acids through first base and/or second base mutation, we conclude that first base and/or second base mutation has relative importance in terms of betweenness centrality.

For degree of centrality point of view we observe that the amino acids R and S have highest degree of centrality. As it does not reflect the indirect link of the amino acids, we cannot draw any conclusion regarding which base mutation represents degree of centrality.

Again when we observe eigenvector centrality it is clear that the amino acids R and S have maximum eigenvector centrality because the sum of direct and indirect links of the amino acids R and S has maximum. As eigenvector centrality depends on direct as well as indirect link, and as indirect link of any of the R and S with other amino acids is through first base and/or second base mutation, we conclude that the first base or third base mutation has relative importance in the context of eigenvector centrality.

### 3.2. Correlation between Various Centralities

In this section we have discussed the bivariate correlation of various measures of centralities for amino acids networks. Correlation is the most important character to study assortative or disassortative networks. A network is called assortative if the vertices with higher degree have the tendency to connect with other vertices that also have high degree of connectivity. If the vertices with higher degree have the tendency to connect with other vertices with low degree then the network is called disassortative. The correlation coefficients for all the centrality measures are shown in Table 2. All correlation coefficients (*r*) are based on Pearson's method. The range of *r*-value is between +1 and −1. If *r* > 0 then the network is assortative whereas if *r* < 0 then the network is disassortative.

From Table 2, we observe that all the centrality measures are highly correlated. Also from the above correlation coefficients we observed that the networks are of assortative type (*r* > 0). Therefore the information can be easily transferred through this network.

## 4. Network Parameters

There are various network parameters which are used in the biological network. In this paper we have used basically three network parameters, namely, clustering coefficient, degree of distribution, and Pearson's skewness. Clustering coefficient is the measurement that shows the tendency of a graph to be divided into cluster. A cluster is a subset of vertices that contains lots of edges connecting these vertices to each other. The clustering coefficient *C*_*i*_ of a node “*i*” is the ratio between the total number (*e*_*i*_) of links actually connecting its nearest neighbours and the total number (the number of such links is *K*_*i*_(*K*_*i*_ − 1)/2, where *K*_*i*_ is the degree of node “*i*”) of all possible links between these nearest neighbours. It is given by *C*_*i*_ = 2*e*_*i*_/*K*_*i*_(*K*_*i*_ − 1). Also nodes with less than two neighbors are assumed to have a clustering coefficient of 0. It takes values as 0 ≤ *C*_*i*_ ≤ 1. The clustering coefficient of the whole network is the average of all individual *C*_*i*_. The higher clustering coefficient of a node represents strong relationship between neighbouring nodes. That is, the higher value of the clustering coefficients of a node represents more number of connections among its neighbours. In Table 3 we have shown clustering coefficients of all the amino acids.

From here it is clear that clustering coefficient of an amino acid depends upon degree of the amino acids as well as number of direct connections between two neighbouring amino acids. Here we observe that the very large hydrophobic amino acid W (volume 227.8 A^3^) as well as high molecular weight (204.23) amino acid has high clustering coefficient. Again the clustering coefficient of whole amino acids network is 0.464 (G). That is, very small hydrophobic amino acid (volume 60.1 A^3^) as well as very small molecular weight (75.07) amino acid represents clustering coefficient of the whole network. Since clustering coefficient is higher with higher number of connections among the neighbours, therefore the higher values of clustering coefficients of a network give large effect on the nodes of the network and slow down the information spread. Therefore from here it is clear that the information can be sent faster in amino acid network.

Next, it is of interest to investigate the nature of the node of the distribution of degrees of nodes for both patterns. The spread in the number of links a node has is characterized by a distribution function *P*(*k*). The degree distribution *P*(*k*) of a network is defined to be the fraction of nodes in the network with degree *k*. If there are *n* nodes in total in a network and *n*_*k*_ of them have degree *k*, we have *P*(*k*) = *n*_*k*_/*n*. Generally the degree distributions value of a node represents the probability that a selected node will have exactly *k* links. In Table 4, we have shown degree of distribution values of different amino acids.

Also another well-known parameter is skewness. Skewness is a measure of the symmetry or asymmetry of the distribution of a variable. The measuring skewness was first suggested by Karl Pearson in 1895. There are various measures of skewness. In this paper we have used only Pearson's coefficient of skewness.In normal curve the mean, the median, and the mode all coincide and there is perfect balance between the right and the left sides of the curve. The situation of skewness which means lack of symmetry occurs in a curve when the mean, median, and mode of the curve are not coincident. Skewness describes the shape of the distribution. Symmetry means that the variables are equidistance from the central value on either side. Again the term asymmetrical means either positively skewed or negatively skewed. Skewness is denoted in mathematical notation by *S*_*k*_. Based on the values and relative position of the mode, mean and median there are two types of skewness that appear in the distribution, namely, positive skewness and negative skewness. If mean is maximum and mode is least and the median lies in between the two then it is called positive skewed distribution. Again if mode is maximum and the mean is least and the median lies in between the two then it is called negative skewed distribution. There are various relative measures of skewness. In this study we have discussed Karl Pearson's coefficient of skewness, which is given by the following formula:

(6) $$\begin{array}{c} S_{k}=\frac{3\left(\text{Mean}-\text{Median}\right)}{\text{Standard}\;\text{deviation}}. \end{array}$$

The value of the measure of the skewness lies within the range of –3 to +3.

If *S*_*k*_ = 0, then the distribution is symmetrical, that is, normal.

If *S*_*k*_ > 0, then the distribution is positively skewed.

If *S*_*k*_ < 0, then the distribution is negatively skewed. Here we assume the degree of distribution as variable (*X*) and number of the amino acids which contains the same value of the distribution as frequency (*f*). Then we have Table 5, where 0.15 is considered as assumed mean.

From Table 5, we have that mode is 0.4 (because of the highest frequency, i.e., 8) and median is 0.294. Also the standard deviation is 0.137. Therefore Pearson's coefficient of skewness is −1.18 < 0. From here we concluded that the degrees of distribution of the amino acids are negatively skewed distribution.

## 5. Conclusion

In this paper we have equipped the amino acids with graph structure by defining compatibility relation based on mutation. We have observed that the graph is connected. We have discussed different centrality measures and we observed that the high hydrophilicity amino acid R (Arginine) and least hydrophilicity amino acid S (Serine) have the highest centrality values irrespective of the centrality measures. Both the amino acids are hydrophilic with the same number of codons, that is, six. The degree of centrality assigns the top score to the amino acids S and R and second top score to the amino acid L, followed by the amino acid I and then the amino acids G, V, and T (fourth score); A, C, D, P, M, K, N, and H (fifth score); E, F, Y, and Q (sixth score); and finally W (seventh score). The only other measure that operates such distinction is closeness centrality. Neither the betweenness centrality nor eigenvector centrality has such distinction. Also we have observed that first base and/or second base mutation has relative importance in all the centrality measures. Next we have found correlation coefficients of the various centrality measures of amino acids and it was observed that all the centrality measures are highly correlated. Hence we can conclude that in amino acid network based on mutation all centrality measures give same ranking to the amino acids. Also we have observed that large hydrophobic amino acid W (Tryptophan) has high clustering coefficient, and small hydrophobic amino acid G (Glycine) has average clustering coefficient of the network. Finally we have observed that the degree of distribution is negatively skewed. Then using Kolmogorov-Smirnov test we observed that the degree of distribution follows three parameter Weibull distribution patterns. Since our graph is based on the mutation of codon, the network show generated gives a general picture of the evolution of the amino acid. An Amino acid *α* has more affinity to evolve from another amino acid say *β* if they are linked than other wise.

## Figures and Tables

**Table 1:** Centrality values for all amino acids.

Vertex	Degree centrality (*C*_*d*_)	Closeness centrality (*C*_cl⁡_)	Betweenness centrality (*C*_bwt_)	Eigenvector centrality (*C*_*λ*_)
S	12	0.730	15.350	0.336
R	12	0.730	14.716	0.342
L	10	0.678	10.100	0.289
I	9	0.655	5.716	0.276
G	8	0.633	7.616	0.219
V	8	0.633	8.166	0.210
T	8	0.633	4.149	0.246
A	7	0.612	4.449	0.191
D	7	0.612	6.166	0.163
P	7	0.612	2.983	0.220
K	7	0.612	4.366	0.198
N	7	0.612	5.533	0.192
H	7	0.612	5.033	0.195
M	6	0.593	3.833	0.143
C	6	0.593	3.833	0.143
E	6	0.593	3.833	0.143
F	6	0.593	2.149	0.183
Y	6	0.593	2.750	0.172
Q	6	0.593	3.283	0.158
W	5	0.575	0.450	0.173

**Table 2:** Correlation coefficients for the centrality positions.

	*C*_*d*_	*C*_cl⁡_	*C*_bwt_	*C*_*λ*_
*C*_*d*_	1	0.998	0.947	0.952
*C*_cl⁡_	0.998	1	0.951	0.951
*C*_bwt_	0.947	0.951	1	0.820
*C*_*λ*_	0.952	0.951	0.820	1

**Table 3:** Clustering coefficient of the amino acids.

G	A	V	L	I	M	P	F	W	Y	N	Q	S	T	C	D	E	K	R	H

0.464	0.476	0.392	0.422	0.472	0.571	0.571	0.6	0.8	0.466	0.428	0.533	0.348	0.5	0.6	0.428	0.466	0.476	0.409	0.428

**Table 4:** Degree of distribution of amino acids.

G	A	V	L	I	M	P	F	W	Y	N	Q	S	T	C	D	E	K	R	H

0.15	0.4	0.15	0.05	0.05	0.4	0.4	0.2	0.05	0.2	0.4	0.2	0.1	0.15	0.4	0.4	0.2	0.4	0.1	0.4

**Table 5:** Calculation of Pearson's coefficient of skewness.

*X*	*f*	*d*_*x*_ = *X* − 0.15	*fd*_*x*_	*fd*_*x*_^2^
0.05	3	−0.1	−0.3	0.03
0.1	2	−0.05	−0.1	0.005
0.15	3	0	0	0
0.2	4	0.05	0.2	0.01
0.4	8	0.25	2	0.5
